# A bibliometric and visualization analysis of electrochemical biosensors for early diagnosis of eye diseases

**DOI:** 10.3389/fmed.2024.1487981

**Published:** 2025-01-10

**Authors:** Fushen Zhang, Weiye Xu, Zejun Deng, Jufang Huang

**Affiliations:** ^1^Department of Anatomy and Neurobiology, School of Basic Medical Sciences, Central South University, Changsha, China; ^2^School of Materials Science and Engineering, State Key Laboratory of Powder Metallurgy, Central South University, Changsha, China

**Keywords:** electrochemical biosensors, eye diseases, tears, Bibliometrics, VOSviewer, CiteSpace

## Abstract

Electrochemical biosensors can provide an economical, accurate and rapid method for early screening of disease biomarkers in clinical medicine due to their high sensitivity, selectivity, portability, low cost and easy manufacturing, and multiplexing capability. Tear, a fluid naturally secreted by the human body, is not only easily accessible but also contains a great deal of biological information. However, no bibliometric studies focus on applying electrochemical sensors in tear/eye diseases. Therefore, we utilized VOSviewer and CiteSpace, to perform a detailed bibliometric analysis of 114 papers in the field of research on the application of tear in electrochemical biosensors screened from Web of Science with the combination of Scimago Graphica and Microsoft Excel for visualization to show the current research hotspots and future trends. The results show that the research in this field started in 2008 and experienced an emerging period in recent years. Researchers from China and the United States mainly contributed to the thriving research areas, with 41 and 29 articles published, respectively. Joseph Wang from the University of California San Diego is the most influential author in the field, and *Biosensors & Bioelectronics* is the journal with the most published research and the most cited journal. The highest appearance keywords were “biosensor” and “tear glucose,” while the most recent booming keywords “diagnosis” and “in-vivo” were. In conclusion, this study elucidates current trends, hotspots, and emerging frontiers, and provides future biomarkers of ocular and systemic diseases by electrochemical sensors in tear with new ideas and opinions.

## 1 Introduction

With increasing emphasis on health care due to the improvement of living standards, early and painless diagnosis of disease is highly required. Non-invasive sensors for specific biomarkers are emerging technologies in the forthcoming era of Digital Medicine. Leland Clark Jr. pioneeringly proposed a glucose oxidase-modified biosensor to achieve electrochemical detection of the biomarker of diabetes (e.g., glucose), paving a new avenue to the biosensor field for early diagnosis of bioanalytes (1). Portable or wearable sensors are gradually coming into the limelight as they offer quicker and more convenient detection methodologies compared to conventional medical methods (e.g., blood tests). A growing number of researchers are working on the development of wearable biosensors interlinked with smartphones or other mobile devices to monitor the body’s health status or disease progression (2).

Biosensors are defined as analytical devices incorporating a biological material, a biologically derived material or a biomimetic intimately integrated within a physicochemical transducer or transducing microsystem (3). In these devices, the working principle of biosensors relies on mutual interactions between target analytes and biometric receptors such as antibodies/antigens, receptors/ligands, and enzymes/substrates, which can generate measurable electrochemical signals to detect target biomarkers in biological samples (4–6).

Compared to invasive blood tests, tears are a fluid naturally secreted by the body that is not only easy to access but also contains a great deal of biological information. In addition to water and electrolytes, tear carries low molecular weight organic compounds, proteins, enzymes, and other biomolecules that can be used for disease screening or early disease diagnosis (7, 8). Tears are considered to be an ideal source of biomarkers because the amount of biomarkers contained in tears varies during different diseases (9). Several studies to date have found that tears contain molecular markers for a wide range of ocular diseases (e.g., diabetic retinopathy, glaucoma, dry eye, postoperative eye trauma) and even systemic diseases (e.g., cancer, Parkinson’s disease, Alzheimer’s disease) and thus tears can be used as a biospecimen for detecting related diseases (10–16). Researchers have developed a variety of highly sensitive biosensors for the detection of different biomarkers in tears.

Bibliometric analysis is a scientific and quantitative research method commonly used in publications that involves the collection, processing and management of data from previous scientific publications to summarize the progress of a research topic and to analyze the contributions of authors, institutions, journals and countries or regions (17, 18). In addition, bibliometric analyses can identify hotspots, emerging trends and knowledge networks in a given field (19). However, few studies have applied bibliometric analyses to the field of ocular electrodynamic sensors.

This study conducted a systematic bibliometric literature analysis of electrochemical biosensors for tear/eye diseases from 2000 to 2023. By analyzing the trends of annual publications and citations, the contribution and cooperation of countries/regions, institutions, authors, journals, and the co-occurrence visualization analysis of keywords, and references, we explored the latest advances in the application of electrochemical biosensors in tears/eye diseases, which aims to fill the knowledge gap between the application of electrochemical biosensors in eye biomarkers, opens the door of electrochemical biosensors in medicine for clinical doctors, and guides researchers to develop new detection devices and technologies. In summary, we aimed to summarize past research and provide a research foundation or new frontier to facilitate further research on eye electrochemical sensors.

## 2 Methods

### 2.1 Data collection and extraction

**Figure 1:**
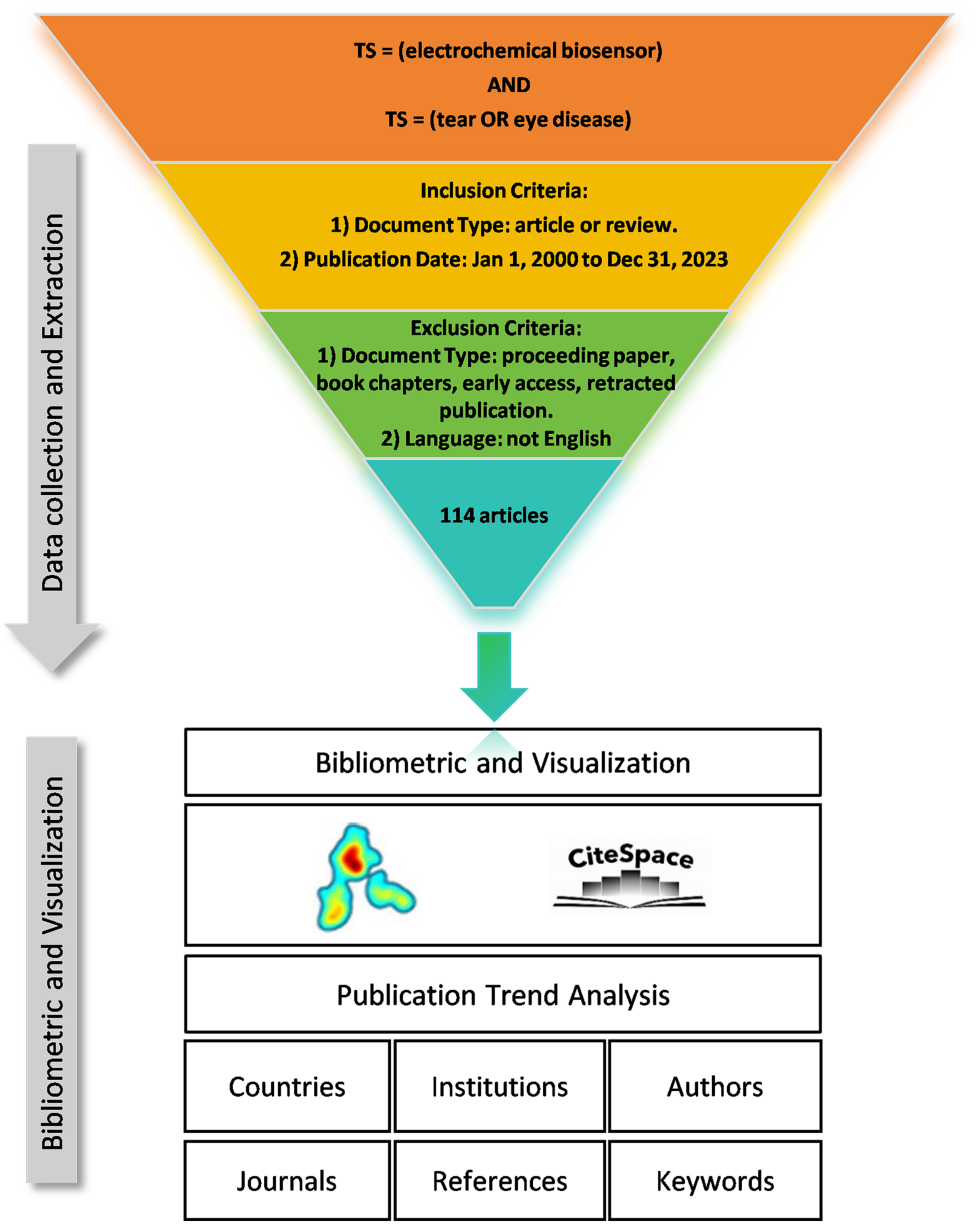
Data screening flow chart and procedures of bibliometric analysis.

We collected literature from the Web of Science Core Collection (WOSCC) database and conducted a literature search on June 6, 2024. The data retrieval strategy and collection process are shown in Figure 1. Briefly, the search strategy was set as “TS = (Electrochemical Biosensor) AND TS = (Tear OR Eye Diseases) AND publication date = (01/01/2000 to 31/12/2023),” then 117 papers were retrieved. The type of publication was restricted to original articles and reviews, and the language was limited to English. Finally, a total of 114 documents were included. This process was executed independently by two researchers, and when disagreements arose, the decision was made by a third researcher.

### 2.2 Bibliometric analysis and visualization

VOSviewer (version 1.6.18), CiteSpace (version 6.1.R6), Microsoft Office Excel (version 2021) and Scimago Graphica were used for literature data extraction and visualization analysis. VOSviewer is a software developed by van Eck and Waltman for bibliometric analysis and mapping (20). In this study, VOSviewer was used for the co-authorship analyses of countries/regions, institutions and authors, the co-citation analyses of journals and authors, and the co-occurrence analysis of keywords. Furthermore, combined with the Scimago Graphica, VOSviewer was utilized for the countries/regions relationship analysis and visualization. In the network map developed by VOSviewer, different nodes represent different elements, such as countries, institutions, authors, journals and keywords. Node size represents the quantity or frequency of elements, while links between nodes reflect the level of collaboration or co-citation. In network visualization, the same color signifies the same cluster. The overlay visualization illustrates the temporal relationship among the nodes. The purple nodes indicate earlier publication times, while the yellow nodes represent more recent ones.

CiteSpace, another widely used bibliometric analysis and visualization tool developed by Professor Chaomei Chen (21), was used to conduct a co-citation network analysis of reference. The citation burst intensities of the references and keywords were also examined using CiteSpace to identify rapidly expanding topic keywords and emerging areas. Moreover, based on the data exported from CiteSpace and the cited report of WOSCC, we used Excel to create an annual publication and citation curve.

## 3 Results

### 3.1 Publication trend analysis

A total of 114 articles were determined in this study, including 67 original articles (86.4%) and 47 reviews (41.23%), the total number of citations was 7,168 times and the average number of citations reached 62.88. This research came from 640 authors in 33 countries and 243 institutions and was published in 74 journals. 8,131 references from 2037 journals were cited.

**Figure 2:**
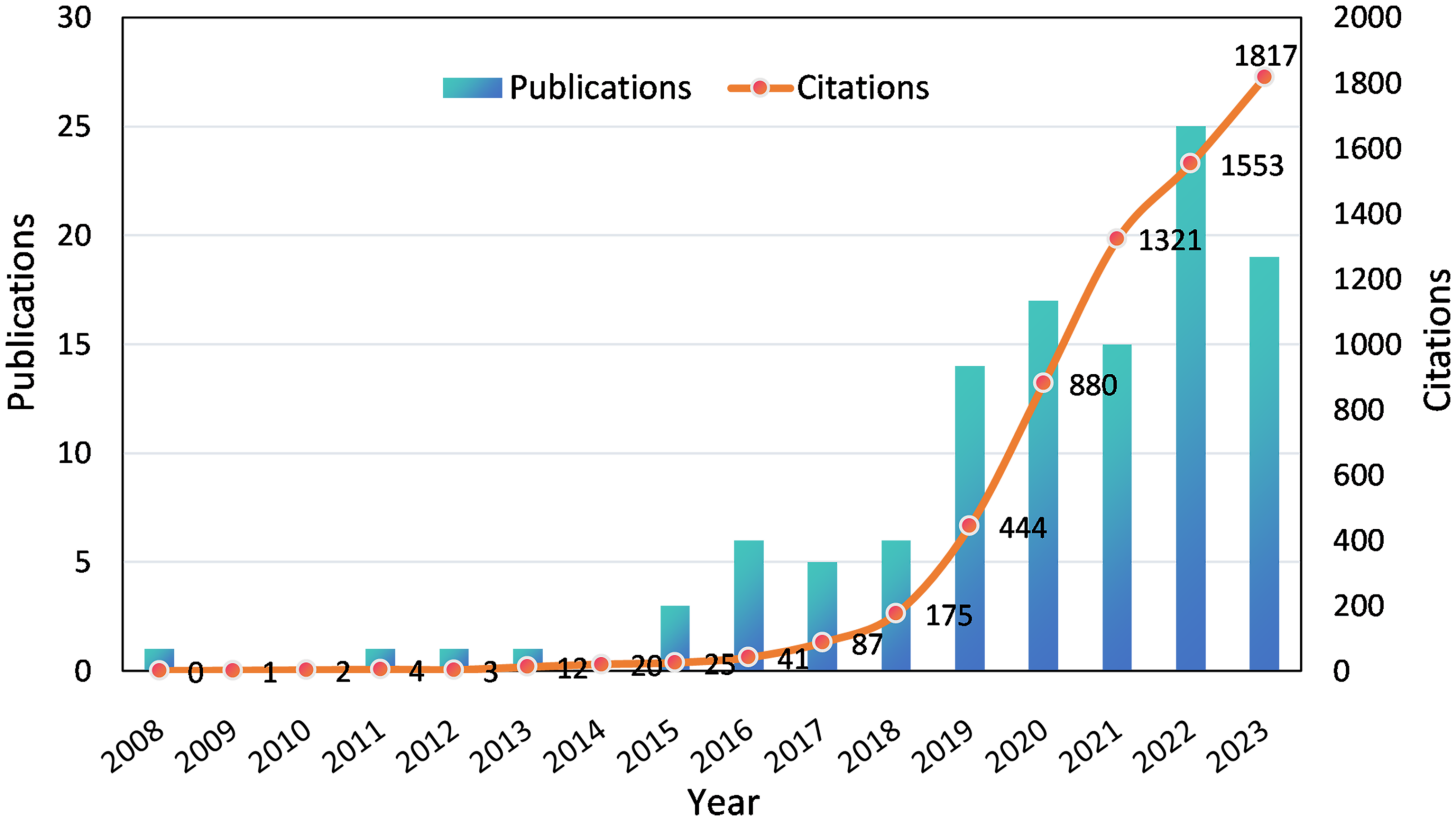
Annual publications and citations on electrochemical biosensors in tear or eye diseases from 2008 to 2023.

Although the inclusion criteria for literature in this study spanned from January 1^st^, 2000 to December 31^st^, 2023, it is noteworthy that the emergence of initial research in this field only occurred after 2008. Furthermore, there has been a consistent upward trajectory since then, with a particularly significant acceleration in growth observed since 2019. Eighty-seven articles were published from 2019 to 2023, accounting for 76.32% of the total (Figure 2). Moreover, there has been a substantial increase in the number of citations to the literature since 2019, particularly within the past 3 years when annual citations surpassed 1,000. This trend signifies an escalating level of attention and significance accorded to this field by researchers.

### 3.2 Countries/regions analysis

**Figure 3:**
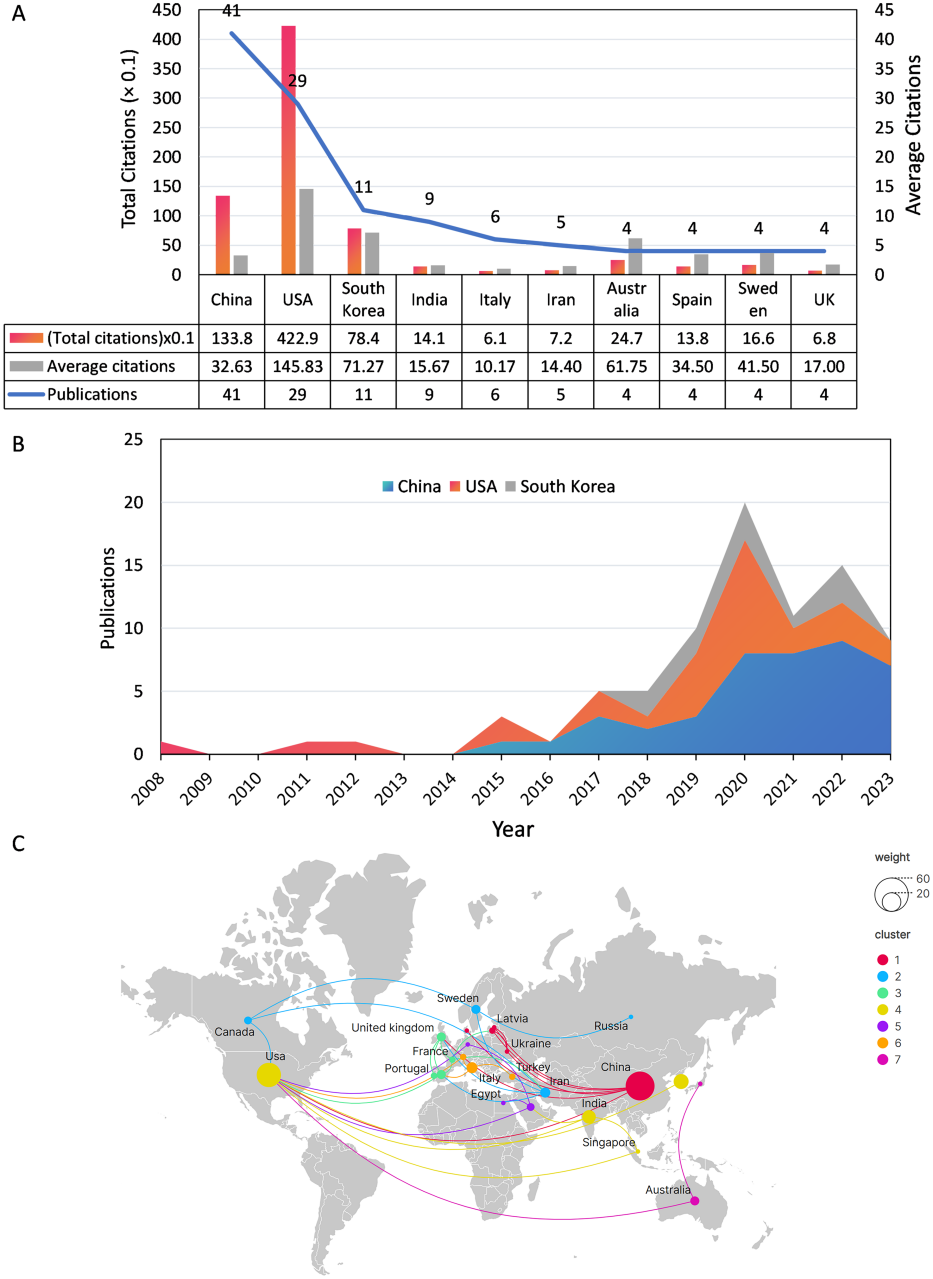
**(A)** Publications, total citations and average citations for the top 10 countries in terms of number of publications. **(B)** Annual output of the top 3 countries with the most publications from 2008 to 2023. **(C)** Map of national co-operation in publishing documents.

Scholars from 33 countries/regions contributed to this field. Figure 3A illustrates the number of publications, total citation count, and average citation count for the top 10 most prolific countries/regions. China is the most prolific country in terms of the number of publications (*n* = 41), followed by the United States (*n* = 29), and South Korea ranks third with 11 publications. It is noteworthy that the United States ranked 2nd in terms of the number of articles published, but it had the highest average number of citations (*n* = 145.8). Conversely, China has the highest number of articles but a lower average number of citations (*n* = 32.6). In addition, Figure 3B shows the annual publication trend of the top 3 countries in terms of publication volume, and it can be seen that all 3 countries will have a significant increase in the number of publications by 2020. Figure 3C depicts the cooperative network relationship among the 25 countries with close cooperation. The United States has the broadest international collaborative network with 10 countries and the highest intensity of collaboration (TLS = 16), which suggests that the USA is more influential in this area of research.

### 3.3 Institutions analysis

**Figure 4:**
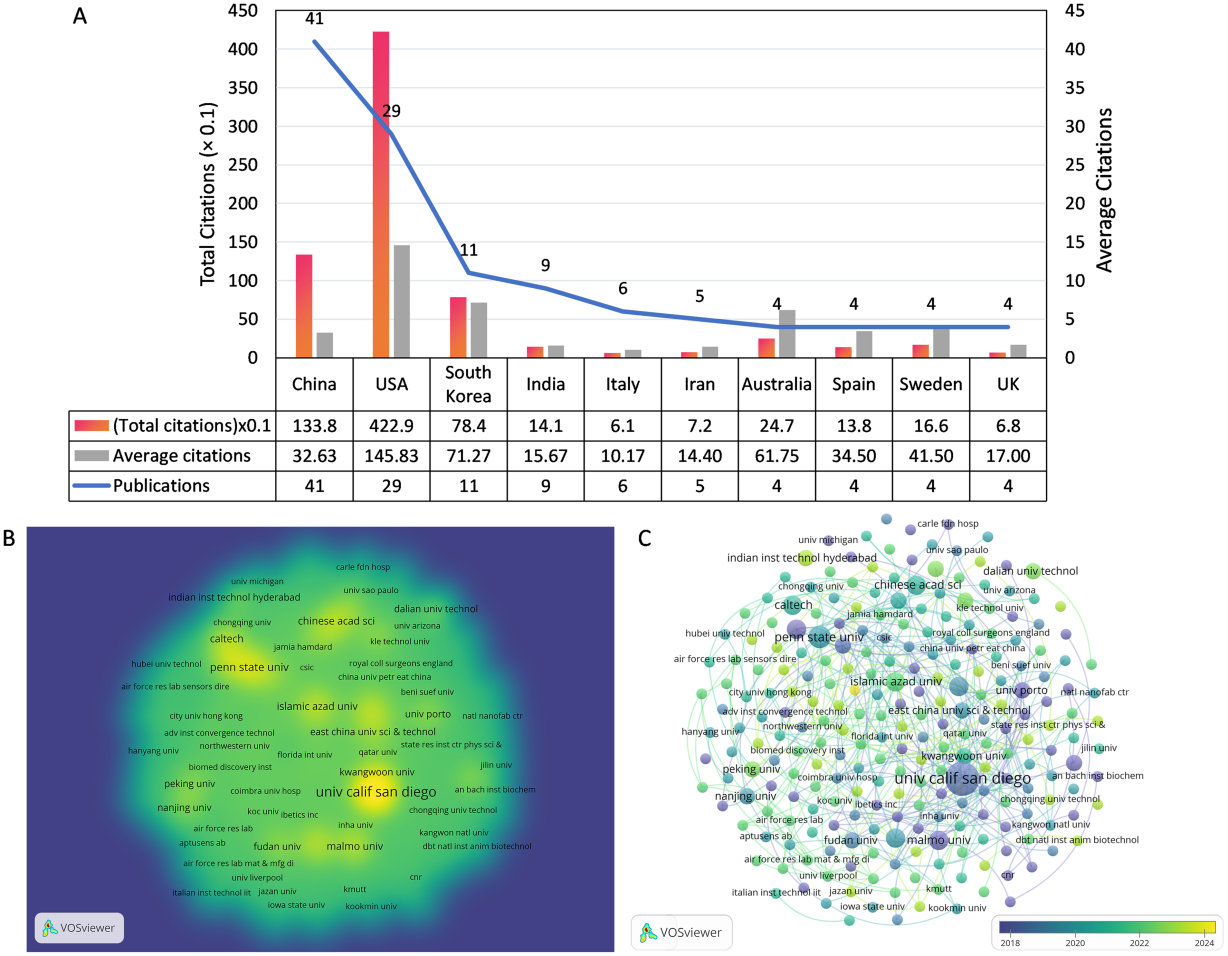
**(A)** The number of publications and citations of the top 10 prolific organizations. **(B)** The co-authorship density visualization map of Institutions. **(C)** The co-authorship overlay visualization map of Institutions.

Two hundred and forty-three institutions were involved in this field, Figure 4A presents the number of publications, total citation count, and average citation count for the top 10 most prolific institutions, and Figure 4B is a density visualization of the number of publications for each institution. The University of California San Diego has the most publications (*n* = 9) as well as the highest total citations (*n* = 246.8), while the California Institute of Technology tops the list with 109.5 total citations and the highest average citations (*n* = 365.0) even though it only published 3 articles. Among the top 10 most prolific research institutions, 4 are located in the United States, 3 in China, and the rest are located in Iran, Sweden, and South Korea, respectively. The overlay visualization of the collaboration network among 243 institutions is presented in Figure 4C. Notably, the University of California San Diego exhibits a high level of collaboration intensity (TLS = 11) with 10 prominent partner institutions.

### 3.4 Authors and co-cited authors analysis

**Figure 5:**
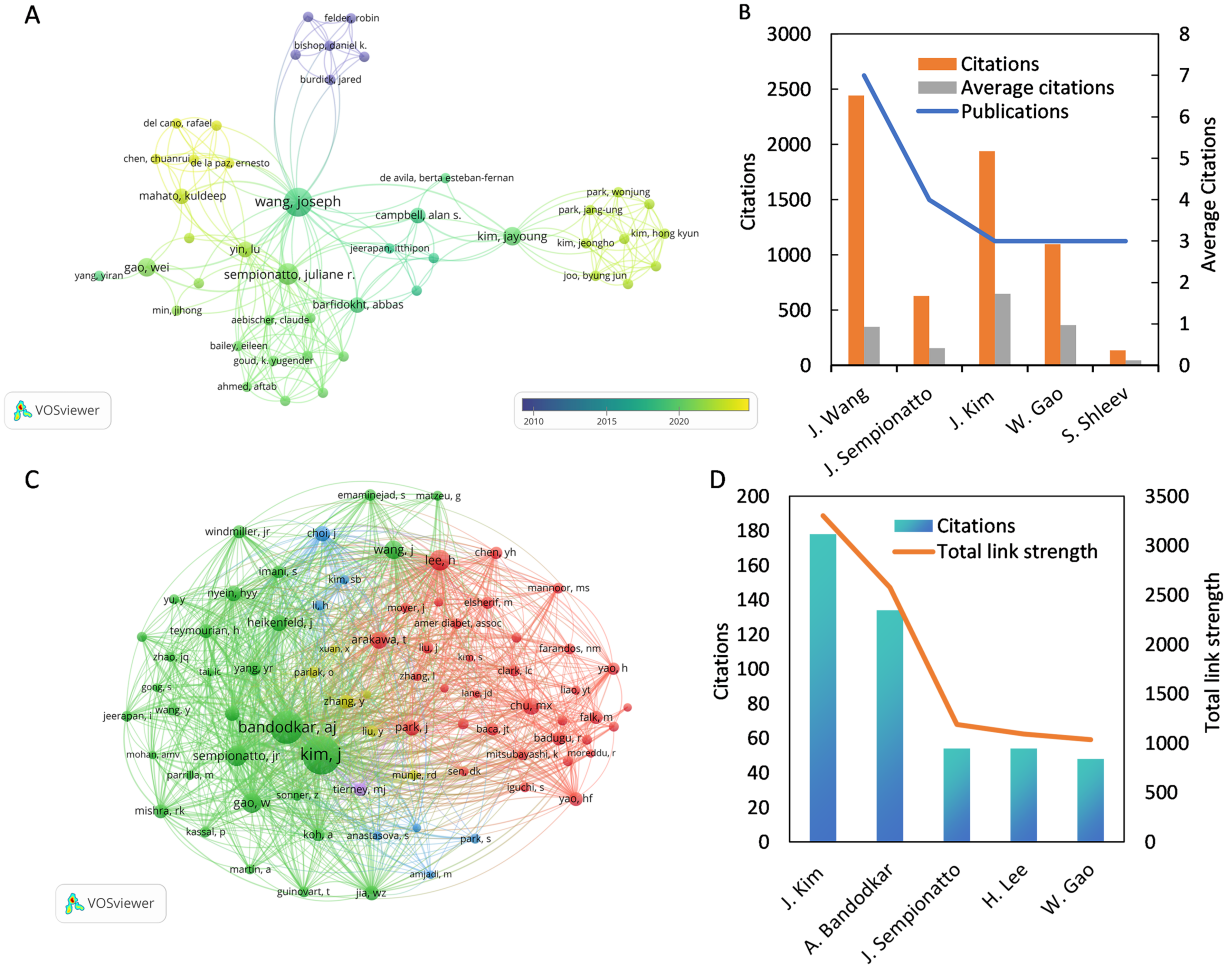
**(A)** The co-authorship overlay visualization map between authors. **(B)** The top 5 of the most prolific authors. **(C)** The co-citation network visualization map of authors related to this field. **(D)** The top 5 of the most cited co-cited authors.

In this field, 640 authors contributed to various studies, with a core group of 45 authors engaging in close collaboration. An overlay visualization was used to illustrate the collaborative interactions among 45 authors, categorized by their average publication year, as depicted in Figure 5A. The yellow nodes represent a more recent average publication year, while the purple nodes indicate an earlier one. As shown in the figure, Chen et al. are the most recent active researchers among the 45 authors, with an average publication year of 2023. Figure 5B and Table 1 show the five most prolific authors, with Joseph Wang leading both categories with seven published papers and 2,443 citations. Joseph Wang also formed collaborations with 34 authors, showing the highest collaboration intensity (TLS = 43). The study involved 6,408 co-cited authors, with 79 authors cited at least 10 times, as shown in Figure 5C. Figure 5D and Table 1 show the five most co-cited authors, with Jayoung Kim from the United States emerging as the most cited author, totaling 178 citations. Despite having only three relevant publications, Jayoung Kim’s work received 1,938 citations, averaging 646 citations per paper, which highlights his significant impact on the field.

**Table 1:** Top 5 prolific authors and co-cited authors on the application of electrochemical biosensors in eye disease research.

Rank	Author	Publications/citations	Institution/Country	Co-cited author	citations	Institution/Country
1	Joseph Wang	7/2443	University of California San Diego (United States)	Jayoung Kim	178	University of California San Diego (United States)
2	Juliane r Sempionatto	4/627	California Institute of Technology (United States)	Amay J Bandodkar	134	Northwestern University (United States)
3	Jayoung Kim	3/1938	University of California San Diego (United States)	H Lee	54	Institute for Basic Science (South Korea)
4	Wei Gao	3/1095	California Institute of Technology (United States)	Juliane r Sempionatto	54	California Institute of Technology (United States)
5	Sergey Shleev	3/137	Malmo University (Sweden)	Wei Gao	48	California Institute of Technology (United States)

### 3.5 Journals and co-cited journals analysis

**Figure 6:**
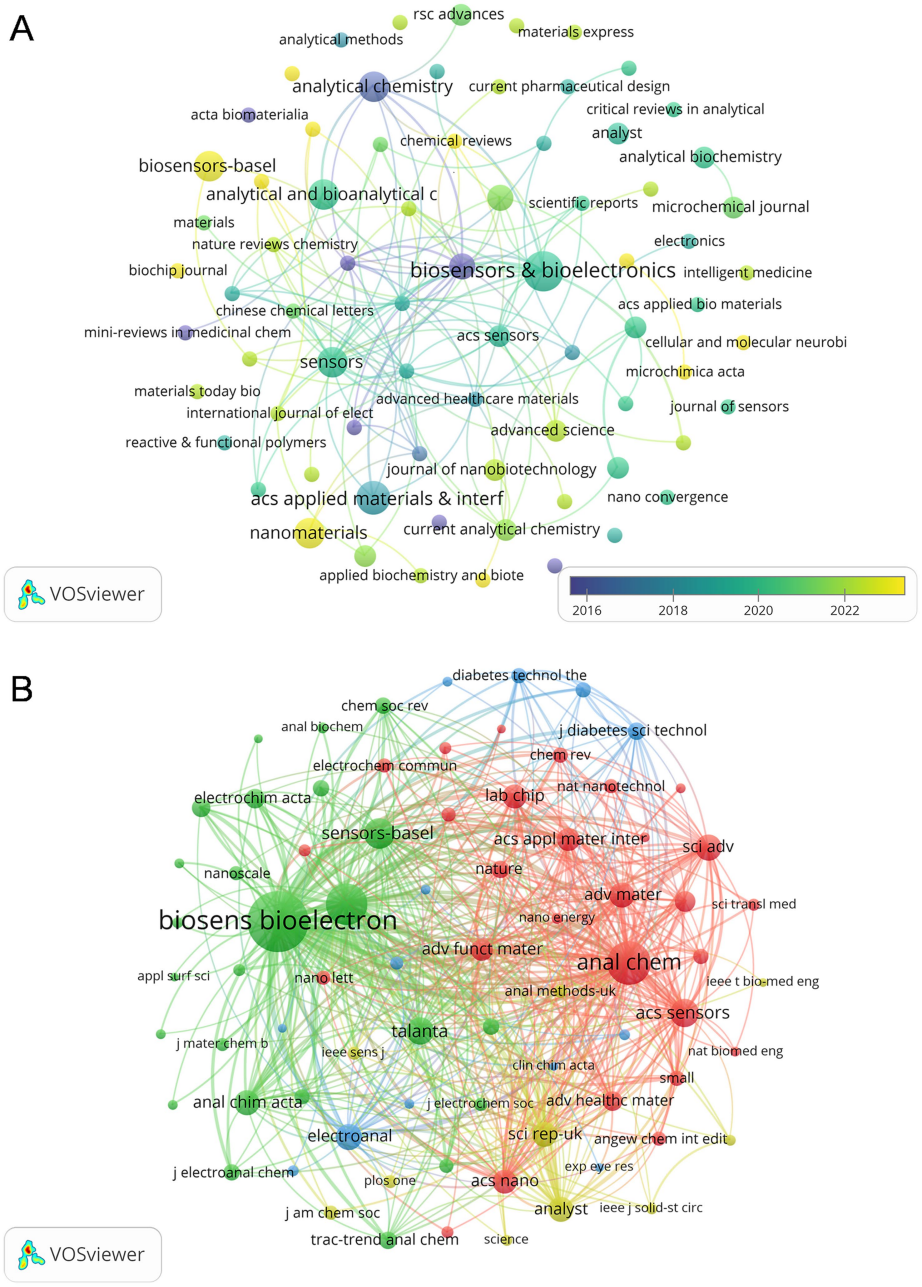
**(A)** The citation overlay visualization map between journals. **(B)** The co-citation network visualization map of journals related to this field.

A total of 74 journals published articles in this research area and Figure 6A shows the overlay visualization of 74 journals for citation analysis. Table 2 provides a summary of the top 10 journals based on publications and co-citations. After sorting by the number of publications, *Biosensors & Bioelectronics* with 7 published articles in the field at the top of the list, and *ACS Applied Materials & Interfaces* is second with 5. In terms of citations, *Biosensors & Bioelectronics* also ranked first with 614 citations, followed by *Analytical Chemistry* (377 citations) and *Sensors* (279 citations). Among them, *Biosensors and Bioelectronics* has the highest impact factor of 12.6 in 2024. Notably, *ACS Sensors* ranked first with an average of 105.5 citations, despite having published only 2 articles in this field. As for the co-cited aspect, there are 2037 co-cited Journals involved in this study, among which 76 journals have 20 co-citations (Figure 6B). *Biosensors and Bioelectronics* leads significantly with a count of 888 co-citations, which is far ahead of the second-ranked *Analytical Chemistry* (454 co-citations), and *Sensors and Actuators b-Chemical* ranks third with 419 co-citations.

**Table 2:** Top 10 productive journals and co-cited journals on the application of electrochemical biosensors in the eye diseases research field.

Rank	Journal	Publications	Citations	Average citations	IF (2022)	Co-cited journal	Co-citations	IF (2022)
1	Biosensors and Bioelectronics	7	614	87.71	12.6	Biosensors and Bioelectronics	888	12.6
2	ACS Applied Materials and Interfaces	5	278	55.60	9.5	Analytical Chemistry	454	7.4
3	Analytical and Bioanalytical Chemistry	4	70	17.50	5.4	Sensors and Actuators b-Chemical	419	8.4
4	Analytical Chemistry	4	377	94.25	7.4	Biosensors-Basel	217	5.4
5	Biosensors-Basel	4	53	13.25	5.4	ACS Sensors	192	8.9
6	Nanomaterials	4	54	13.50	5.3	Science Advances	161	13.6
7	Sensors	4	279	69.75	3.9	Electroanal	160	3
8	Electroanalysis	3	162	54.00	3.0	Analyst	158	4.2
9	Frontiers in Bioengineering And Biotechnology	3	45	15.00	9.5	Scientific Reports	154	4.6
10	ACS Sensors	2	211	105.50	8.9	Analytica Chimica Acta	152	6.2

### 3.6 Citing articles and co-cited references analysis

Out of the 114 articles included, Table 3 lists the top 10 most cited articles. Among them, a review entitled “wearable biosensors for healthcare monitoring” was published in Nature Biotechnology by Wang et al. in 2019, which has been mostly up to 1759 times by 20th July 2024 (2). This review provides a comprehensive summary of the recent advances in wearable biosensor technology and identifies key scientific and technical issues that need to be addressed in the future, providing directional guidance for the further development of the field.

**Table 3:** Top 10 most cited papers on the application of electrochemical biosensors in eye disease research.

Rank	Title	Type	Citations	Journal	IF (2022)	Corresponding author	Affiliation	Year
1	Wearable biosensors for healthcare monitoring (2)	Review	1713	Nature Biotechnology	46.9	Joseph Wang	University of California San Diego	2019
2	Wearable and flexible electronics for continuous molecular monitoring (90)	Review	819	Chemical Society Reviews	46.2	Wei Gao	California Institute of Technology	2019
3	Enzyme-based glucose sensor: From invasive to wearable device (88)	Review	465	Advanced Healthcare Materials	10	Dae-Hyeong Kim & Taeghwan Hyeon	Institute for Basic Science	2018
4	Wearable electrochemical biosensors in North America (91)	Review	142	Biosensors and Bioelectronics	12.6	Joseph Wang	University of California San Diego	2021
5	Body-interfaced chemical sensors for noninvasive monitoring and analysis of biofluids (146)	Review	64	Trends In Chemistry	15.1	John A Rogers	Northwestern University	2019
6	Recent developments of flexible and stretchable electrochemical biosensors (147)	Review	48	Micromachines	3.4	Huanyu Cheng	Chongqing University	2020
7	Wearable electrochemical glucose sensors in diabetes management: A comprehensive review (148)	Review	47	Chemical Reviews	62.1	Joseph Wang	University of California San Diego	2023
8	Non-invasive electrochemical biosensors operating in human physiological fluids (149)	Review	20	Sensors	3.9	Sergey Shleev	Malmo University	2020
9	Current development in wearable glucose meters (150)	Review	17	Chinese Chemical Letters	9.1	Yan Zhao	Fudan University	2021
10	Soft, disruptive and wearable electrochemical biosensors (151)	Review	9	Current Analytical Chemistry	1.8	Wenlong Cheng	Monash University	2022

**Figure 7:**
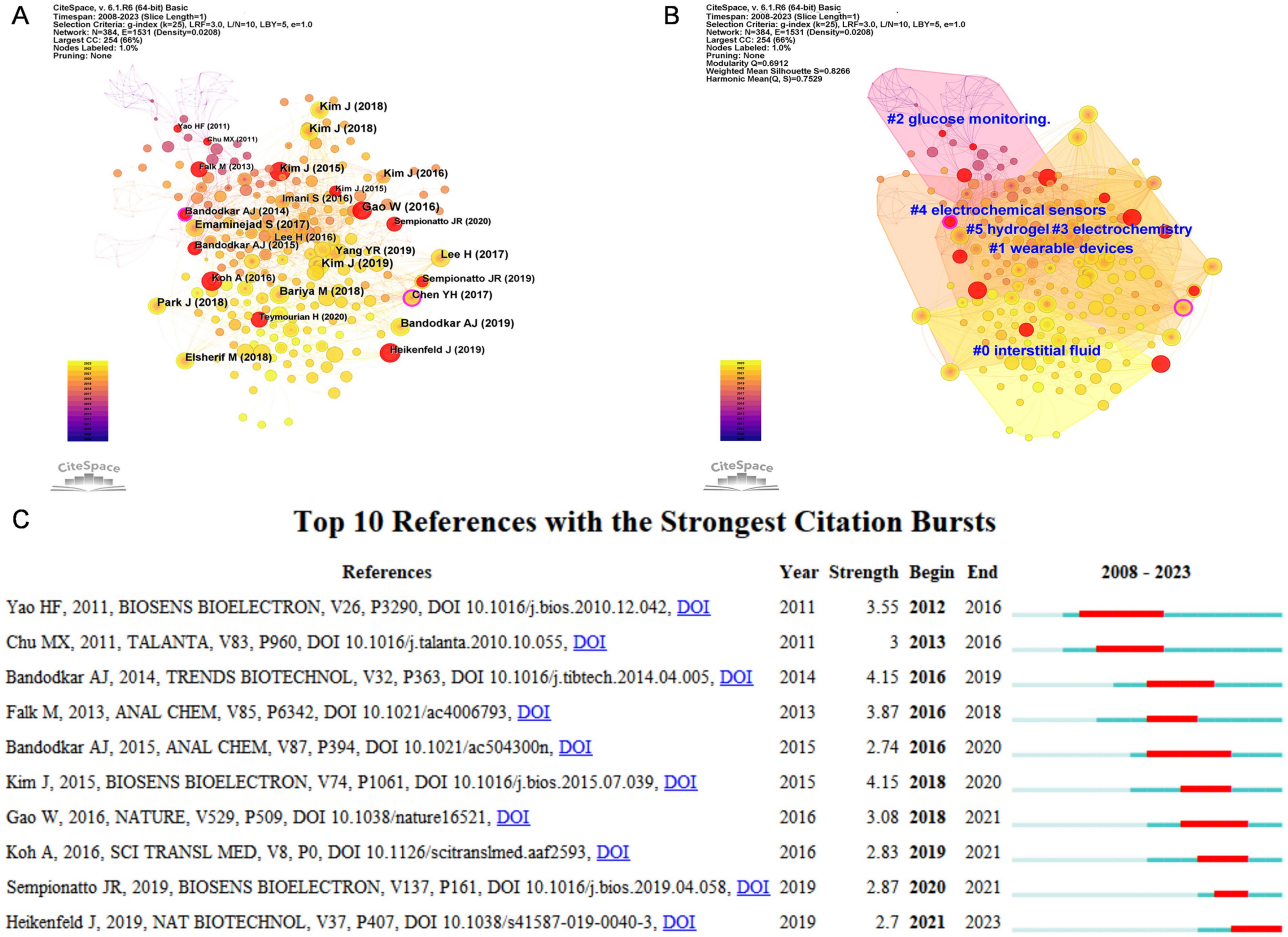
**(A)** Co-occurrence analysis network view of co-cited reference based on CiteSpace. **(B)** Cluster analysis network view of co-cited reference based on CiteSpace. **(C)** Top 10 references with the strongest citation bursts. The blue bar showed that the reference was rarely cited, and the red bar indicated frequent citations.

Two or more papers that are simultaneously cited by one or more papers are said to constitute a co-citation relationship (22). Based on CiteSpace, we created a co-occurrence analysis network (Figure 7A), cluster analysis network (Figure 7B), and burst analysis of co-cited references (Figure 7C). In Figure 7A, the outer rings of Bandodkar AJ (2014) (23) and Chen YH (2017) (24) nodes are in purple, indicating high betweenness centrality and playing a pivotal bridging role (25), emphasizing their instrumental role in facilitating collaboration in the field of electrochemical biosensors and tears/eye disease research. In our co-cited reference clustering analysis, the Modularity Q value reached 0.6912 (>0.3), which implied the robustness of the clustering structure. Moreover, the weighted mean silhouette score reaches 0.8266 (>0.7), which implies a high degree of consistency among the members of each cluster (26). Six clustering results are presented in Figure 7B, “interstitial fluid,” “wearable devices,” “glucose monitoring “, “electrochemical,” “electrochemical sensors,” and “hydrogel,” these clusters reflect the research frontiers in the field of ocular biosensors.

A co-citation burst refers to a citation that appears with high frequency in an article published within a condensed timeframe, indicating increased interest and relevance within the research community. The red line indicates the duration of the burst and the blue line indicates the time from the appearance of the term to its end. Figure 7C shows the details of the top 10 bursts of co-cited literature. Yao HF (2011) (27) and Bandodkar AJ (2015) (28) show the longest duration of bursts, while Kim J (2015) (29) and Bandodkar AJ (2014) (23) have the highest burst intensity.

### 3.7 Keywords analysis

**Figure 8:**
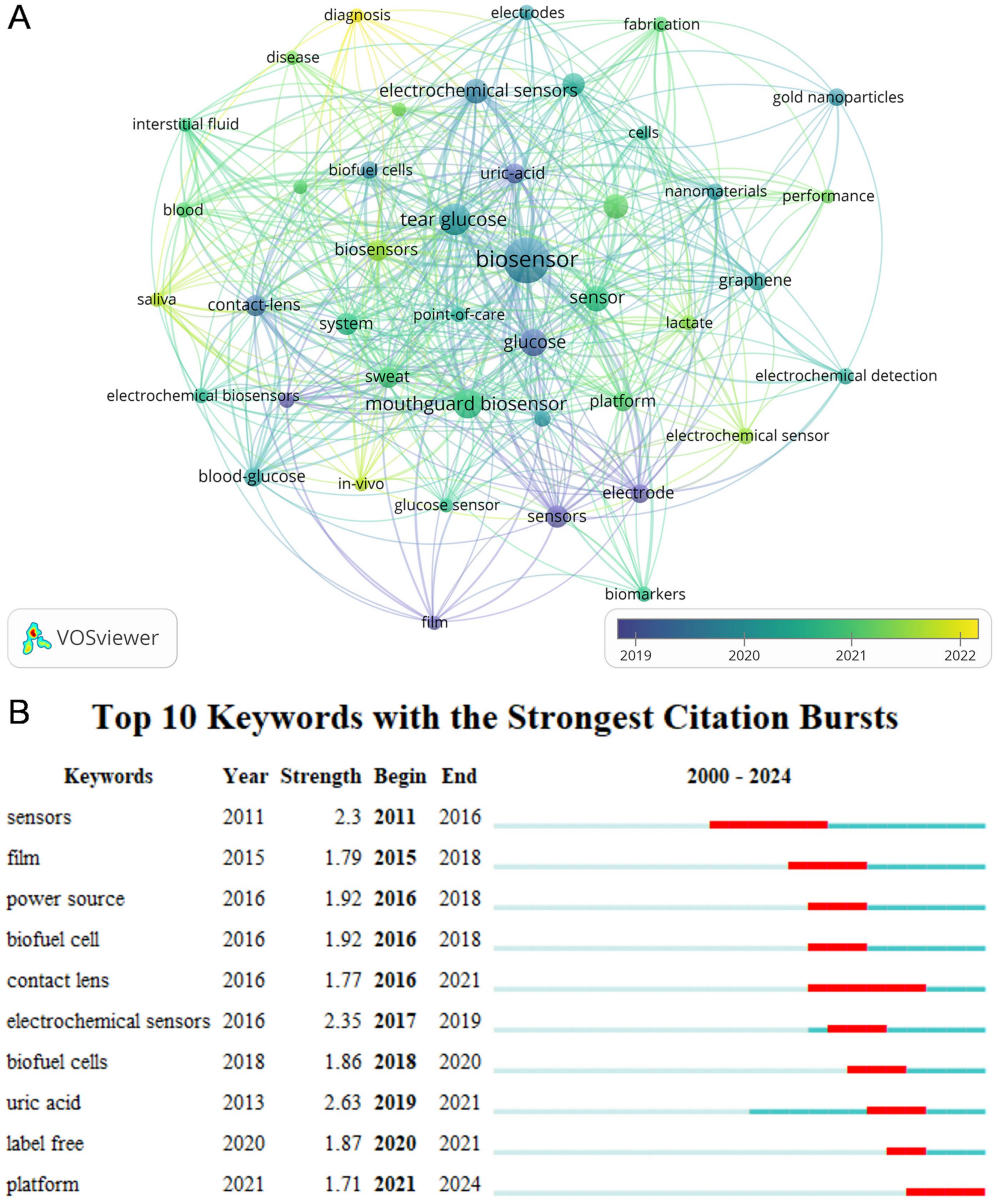
**(A)** The co-occurrence overlay visualization map between keywords. **(B)** Top 10 keywords with the strongest citation bursts. The blue bar showed that the keyword was rarely cited, and the red bar indicated frequent citation.

A co-occurrence analysis of all keywords was performed using VOSviewer to identify impacts and trends within the field, which includes a total of 792 keywords, Figure 8A shows an overlay visualization of 43 keywords that appeared at least five times. Interconnections between the keywords denote co-occurrence, with purple denoting earlier appearing keywords, and yellow representing more recent ones. This network diagram illustrated that the keyword “biosensor” has the highest frequency of occurrence (*n* = 50), with an average appearance year (AAY) of 2019. “Tear glucose” (AAY = 2020) follows as the second most frequent (*n* = 24). Notably, “diagnosis” (AAY = 2022) and “in-vivo” (AAY = 2021) are recent keywords with at least five occurrences, indicating emerging interests in this research domain. To further understand the evolution of research topics, we utilized CiteSpace to examine keyword bursts. Figure 8B illustrates the top 10 keywords with the highest burst intensity, where “sensors” and “contact lens” have the longest burst duration, spanning over 5 years, and “uric acid” exhibits the highest burst intensity.

## 4 Discussion

We analyzed in detail the literature on Electrochemical sensors for eye diseases and tears between 2000 and 2023. We found that the field first appeared in 2008 and has only been in development for 15 years. Starting in 2019, the field has seen a significant acceleration in the number of annual publications and the number of annual citations, suggesting that this field will still be a focus for researchers in the future. In summary, we believe that this area will become a hotspot for research in the future (30). With the development of technology, this fast, convenient, non-invasive analytical device provides more valuable assistance in clinical diagnosis (31, 32). To verify this conclusion, we analyzed in depth 114 papers published between 2000 and 2023. The literature came from 33 different countries, 243 institutions, and 74 journals, with a total of 640 authors covering 792 keywords. These data suggest that the study of tears as electrosensors is a completely new field. As we delve deeper into this field, we find that research on electrochemical biosensors in the eye, although relatively recent, is gaining momentum in an unusually rapid manner. Advances in technology have not only provided new solutions for the early screening of diseases but also brought breakthroughs in the diagnosis and treatment of eye diseases. In the past 5 years, China and the United States of America have published a large number of articles in this field, accounting for 49.12 percent of all articles published. Among the top 10 institutions in terms of publications, four are from the United States and three are from China, and the research institutions in both countries not only have a high volume of publications but also are internationally recognized for the quality of their research. Of these, the University of California San Diego is the most prominent contributor with the highest number of publications and total citations. In terms of journals, *Biosensors & Bioelectronics* leads the field in terms of publications and citations, which focuses on research, design, development, and applications and has a high academic reputation.

We used VOSviewer software to analyze the bibliometrics, and the keywords “biosensor” and “tear glucose” appeared most frequently, reflecting the hotspots and focuses of research in this field. The terms “in-vivo” and “diagnosis” are emerging keywords that signal future directions and trends in the field. In addition, the explosive growth of “sensors” and “contact lenses” also provides us with new research directions and ideas.

### 4.1 Tear biomarkers

In the early screening and diagnosis of diseases, highly sensitive and convenient tests can help us predict the future course of a condition and accurately assess a patient’s health. With the continuous development of electrochemical biosensors, the medical field is witnessing a new type of detection method. This technology enables the detection of samples by collecting and filtering multiple biological samples and integrating them into a chip (33). With the advantages of high sensitivity, low cost, and non-invasiveness, this technology brings great convenience to patients and healthcare professionals. The application of electrochemical biosensors in medicine has achieved remarkable results from the initial detection of glucose content (1, 34, 35), to applications in a variety of fields such as the detection of pathogenic microorganisms (36), diagnosis of respiratory diseases (37–39) and screening of cancer (40–42).

In this field, research using tears as the biomarkers of electrochemical sensors is of particular interest. Tears are a fluid naturally secreted by the human body, which is not only easily accessible but also contains a great deal of biological information. Tear is a meaningful biomarker source and specific diseases showed distinguished proteomic patterns in tears. Tears can be considered an ideal biomarker source because the body is exposed to different pathophysiological conditions during disease periods and the tear content changes accordingly (9). According to proteome analysis of tears, this fluid has approximately 500 proteins; on the other hand, plasma may contain up to 10,000 proteins (43). The composition of its proteins also varies with the disease (44–47). Despite the differences in the components between blood and tears, a portion of biomolecules in the blood can still diffuse and enter into the tear, which probably shows a close correlation between blood and tears. Thus, the biomarkers in tears can be also used to a certain extent to replace those in the whole body, which provides a promising potential for the diagnosis of ocular diseases (8). In addition, the components of tears are not as complicated as blood and contain a variety of trace elements (e.g., potassium, manganese, chloride and sodium ions), biomolecules (e.g., glucose, urea), gamma globulin (e.g., IgA, IgG and IgE) and enzymes (e.g., lysozyme), which can act as vital biomarkers for the diagnosis of diseases and make tears an attractive diagnostic biofluid (43, 48).

By detecting specific substances in tears, we can achieve rapid, non-invasive diagnosis of a wide range of ocular and systemic diseases. Many novel protein-based biomarkers have been correlated to specific ocular diseases, namely dry eye syndrome, trachoma, glaucoma, keratoconus, allergic conjunctivitis, allergy with corneal lesions, blepharitis, conjunctivochalasis, mycotic keratitis, pterygia, thyroid-associated orbitopathy, etc., and systemic disorders, namely diabetes mellitus, cancer, systemic or multiple sclerosis, cystic fibrosis, Parkinson’s disease, sclerosis, etc. (9, 23, 49). Many methods have been used to identify quantitative changes in protein expression in a biological system. Relatively speaking compared to other body fluids, tears are a unique source of protein. Many researchers have therefore focused on changes in the proteomic, lipidomic and metabolomic composition of the tear film during disease.

### 4.2 Detection techniques of electrochemical sensors

For the different substances in tears, the methods used to detect them vary. For certain metabolites in tears, such as glucose, lactate and uric acid, an enzymatic amperometric assay is commonly used to detect (50, 51). For proteins and small molecules in tears, voltammetry using bio-affinity recognition is generally used to detect the target substances (52–55). For the detection of ionic components such as the electrolytes Na^+^, K^+^ and Cl-in tear, the presence of the target substance is detected by measuring the potential signal associated with a change in surface charge at the sensing electrode, using a potentiometric biosensor with a high degree of particle selective penetration (51, 56–59). Among the most commonly used methods of tear sensors for contact lens detection are organic electrochemical transistors (OECT) biosensors and electrochemiluminescence (ECL) sensors. OECT sensors not only have the advantages of low cost, high sensitivity, and low operating voltage (<1 V), but they can also detect a wide range of electroactive substances (e.g., dopamine, glucose, and epinephrine) (60, 61), as well as non-electroactive substances (e.g., cortisol, deoxyribonucleic acid (DNA), proteins, etc.) (62–64). ECL sensors combine the advantages of optical and electrochemical analyses to quantify target molecules by electrochemically emitting different light to emit signals that are associated with target organisms (e.g., glutathione, lactate, DNA, microRNA) (65–67)to identify the luminescence-inducing active substances, and electrochemiluminescence bioimaging to perform cellular Analysis. Electrochemiluminescent cellular bioimaging has been carried out for different small molecules released in cells and for membrane protein targets on the cell surface (68–70). The application of these techniques has led to a deeper study of the tear.

### 4.3 Sensory materials and functional modification

In addition to water and electrolytes, tears carry low molecular weight organic compounds, proteins, enzymes, and other biomolecules that can be used for disease screening (7). In the application of biosensors, electrochemical sensors have shown unique advantages in detecting biological components in tears. Generally speaking, most biomarkers should be integrated into the biosensors by functional modification such as oxidoreductases, antibodies/antigens, or enzymes, which aims to improve the selectivity and sensitivity of the biosensors for a specific biomarker (27, 71–73). Concerning the detection of proteins and organic small molecules, electro-active materials (e.g., enzymes, nanomaterials and electro-active molecules) need to be additionally modified to the biosensor interface since those proteins and organic molecules are usually electrochemically inactive and cannot directly produce electrochemical signals (74–77). Several typical electrode materials widely used by researchers include precious metals such as silver, gold, and platinum, as well as diverse carbon-based materials such as non-metallic carbon materials including graphene, graphite, carbon nanotubes, and glassy carbon (78). Different carbon substitutes are used for different tear biomarkers to achieve sensitive modulation of electrode performance, thus achieving precision and accuracy of detection (79). In addition, to further enhance the performance of electrochemical sensors, researchers often employ polymers as modifying materials for the electrodes and finely modify the electrode surfaces by introducing nanofibres or nanoparticles, etc., to effectively improve the sensitivity and responsiveness of the sensors (78, 79).

### 4.4 Tears-based electrochemical biosensors

A glucose oxidase-modified biosensor, pioneered by Leland Clark Jr. in 1960, enabled electrochemical detection of a diabetes biomarker (i.e., glucose), opening up a new avenue for early diagnosis of analytes in the biosensor field (1). The use of an electrical biosensor device inserted into the lacrimal gland to detect glucose and health-related biomarkers in tears was first proposed in 2008 by Joseph Wang of the University of California, San Diego, as an alternative to traditional blood sampling (80). Yan et al. developed an electrochemical sensor with low sampling volume, good selectivity, and high reproducibility, which enabled the first noninvasive detection of glucose in rabbit tears (81). In subsequent studies, more and more researchers have tried to change the electrode materials and compositions by, for example, using graphene electrodes and silicon oxide electrodes (82), nanoporous gold electrodes, etc., to improve the sensitivity and specificity of the assay and enable more accurate identification of components in tea (83–85). To achieve a more convenient and non-invasive monitoring method, Yao et al. from the University of Washington developed a contact lens that is non-invasive and allows continuous monitoring of tear glucose levels over a long period (86). With the advent of these electrochemical sensing contact lenses, we have transitioned from traditional invasive and invasive testing to a non-invasive, portable and wearable era. This breakthrough undoubtedly opens up unlimited possibilities for the application of biosensors in medicine (87–89).

Researchers and academics are convinced that smart wearables will inevitably play a central role in the future of healthcare (2, 90–92). This trend has been widely recognized and a large number of researchers have begun to focus on contact lenses. Contact lenses are more than just a fashion accessory; they can monitor a variety of biomarkers through tears, providing important information for disease diagnosis (8). Since early studies, scientists have explored techniques for detecting glucose in tears (1), and to this day we have been able to accurately detect amyloid Aβin tears using small, non-invasive biosensors. Remarkably, the concentration of Aβ in tears is approximately 10 times higher than the concentration of Aβ in blood, making it an important indicator of early diagnosis of Alzheimer’s disease (93, 94). Song et al. explored the relationship between tears and blood cholesterol levels using a rabbit model of hyperlipidemia and successfully used contact lenses with electrochemical sensors to detect the feasibility of cholesterol-associated disorders in tears, a technique that not only provides a non-invasive diagnostic method but also has the potential to bring about a much more convenient healthcare experience for patients (95). In addition, the researchers found that the amount of urea in tears was comparable to that in blood, which provides a more effective way to test kidney function (8, 96). Also, the concentration of TNF-*α* in tears can be monitored by a rapid and simple assay, which is important for early screening and assessment of cancer (97, 98). The detection of cortisol has also been a hot topic in studies exploring tears as a biomarker; most cortisol is released from the adrenal glands where it binds to corticosteroid-binding globulin, but there is also an active form of free cortisol that occurs in biological fluids, including tears, a finding that provides a new way to monitor and assess the body’s stress response (99). Sempionatto et al. achieved the detection of vitamin C in tears by immobilizing ascorbic acid oxidase on a flexible printable tattoo electrode (100). This innovative technology not only provides a new means of health monitoring but also offers a deeper understanding of tears as a biomarker. Overall, smart wearable devices have great potential for future applications in medicine. By detecting various biomarkers in tears, these devices will be able to provide important information for the early diagnosis and treatment of diseases, thus protecting people’s health.

With the continuous development of science and technology, more and more researchers are focusing their attention on the combination of electrochemical biosensors with wireless smart devices for real-time visual monitoring (10). Kim et al. utilized enzyme-functionalized graphene-silver nanowire hybrid nanostructures to monitor glucose concentration in tears and intraocular pressure (IOP) through contact lenses with integrated glucose sensors, wireless power transmission circuits, and display pixels inside the lens (101). Later, researchers at Yonsei University in South Korea developed contact lenses capable of real-time cholesterol monitoring (95). In addition, significant progress has been made in research on contact lens-based biosensors for optical monitoring, Elsherif et al. developed an electrochemical sensor that produces a change in the volume of the hydrogel when the glucose concentration changes thereby changing the intensity of the diffracted light and synchronizes the information to a smartphone for monitoring (102). Ye et al., development of a contact lens for detection of matrix metalloproteinase-9 (MMP-9) and intraocular pressure in eye-related diseases by surface-enhanced Raman scattering and color change, respectively (13). Sempionatto et al. developed an electrochemical sensor that can simultaneously monitor the levels of three biomarkers, glucose, alcohol and vitamins, in tear (103). More and more researchers are committed to expanding the application scenarios of electrochemical sensors to the home healthcare industry, and these sensors will be closely connected with smartphones to provide patients with a full range of medical information and protection, which is undoubtedly research of great significance and is expected to change the way of life of people’s healthcare and make healthcare services more efficient and convenient.

### 4.5 Typical eye diseases detected by electrochemical biosensors

#### 4.5.1 Diabetic retinopathy

Diabetic retinopathy (DR) is one of the most serious ocular microvascular complications of diabetes mellitus and a major cause of vision loss in patients (104–106). The number of people with DR is expected to increase to 119 million globally by 2030 (107). This not only poses a serious threat to the quality of life of people with diabetes but also imposes a heavy financial burden on social healthcare coverage (107). Effective control of blood glucose can slow down the progression of DR, making monitoring blood glucose a must for diabetics. Although there are more convenient and quicker portable blood glucose meters for home use, the use of this instrument does not allow continuous monitoring of blood glucose changes in real-time and requires strict sterilization to detect the blood glucose concentration of fingertip blood. As a result, a large number of researchers have developed a variety of sensors that use tears to monitor blood glucose (108, 109). Early screening and prompt treatment can prevent diabetes-related eye complications, but screening and diagnosis of DR are not well established (110). Kim et al. found that lipocalin-1 (LCN-1), heat shock protein 27, and beta-2 microglobulin expression in tears contributed to the diagnosis of DR patients (111). Furthermore, Costagliola et al. suggested that there is a relationship between high levels of tumor necrosis factor alpha (TNF-*α*) and DR severity in DR patients (112). It has also been suggested optoelectronic and optoelectrokinetic bead-based immunosensors for lipocalin-1 detection (11, 12) and Au-NP-doped diffusometric immunosensors for TNF-*α*detection have been developed to diagnose diabetic retinopathy in tears (113). The proposal of these tear biomarkers provides new research avenues for the use of tear sensors for screening and diagnosis of DR.

#### 4.5.2 Dry eye syndrome

Dry eye syndrome (DES) is a multifactorial ocular disease characterized by tear film instability and disruption of ocular surface homeostasis, with pathogenic mechanisms including ocular surface inflammation and injury (114–116). DES is also one of the common eye complications in diabetics, with more than 50 percent of diabetics also being diagnosed with DES, and there is a clear relationship with the duration of diabetes (117–119). Therefore Han et al. concluded that monitoring blood glucose levels and DES would be beneficial for diabetic patients, and for this reason, the team developed a non-invasive anterior eye sensor system for monitoring diabetes mellitus and dry eye syndrome (109). Considering the association of DES with inflammation, cytokines and chemokines have also been identified as potential targets for DES diagnosis, such as Interleukin-1, Interleukin-6, MMP-9, TNF-α, Interleukin-17A and Interleukin-1RA (120–124). One of these has been developed as a commercially available point-of-care based on the inflammatory protein MMP-9, a device that can show the degree of inflammation in DES of MMP-9 (125, 126). Zhou et al. Using proteomics techniques, the identified the up-regulation of α -enolase, α -1 acid glycoprotein 1, calgranulin A, calgranulin B, and calgizzarin, in addition to four down-regulated proteins, prolactin-inducible protein, LCN-1, lactoferrin, and lysozyme in the DES patients (127). In addition, researchers have attempted to accurately diagnose DES using biomarkers in lipidomics and metabolomics (128, 129). The study of these biomarkers provides a better research direction for the development of electrical biosensors for applications in tears.

#### 4.5.3 Glaucoma

Glaucoma is a group of optic neuropathies characterized by progressive degeneration of retinal ganglion cells and is the leading cause of irreversible blindness worldwide (130, 131). Glaucoma is a multifactorial disease whose pathogenesis is not fully understood, but elevated intraocular pressure is the only proven modifiable risk factor associated with the development of optic nerve damage in glaucoma (130, 132, 133). Detection of IOP is therefore important for the diagnosis and prediction of prognosis in patients with glaucoma. Measurement of IOP is currently achieved mainly using tonometers, which usually need to be performed in clinics or hospitals and do not facilitate real-time monitoring of IOP in glaucoma patients. For sensors to detect IOP, the SENSIMED Triggerfish from Sensimed AG (Switzerland) is a single-use soft contact lens that measures IOP-related changes in ocular dimensions and is currently CE marked to Class IIa (8). A related study reported that MMP-9 was overexpressed in patients with early glaucoma (134), and Ye et al. developed an ocular sensor that simultaneously monitors IOP and MMP-9 levels in tears (13). Zubareva et al. suggest that tear-based non-invasive testing for catecholamines can improve the diagnosis of glaucoma (135). Joachim et al. suggested that in the future, the application of auto-antibody reactivities (HSP10, HSP27, myelin basic protein and Protein S100) (136) in tear for electrochemical sensors will provide a better method of assessing early screening and diagnosis of glaucoma.

#### 4.5.4 Age-related macular degeneration

Age-related macular degeneration (AMD) is a chronic progressive neurodegenerative retinal disease that, if not treated appropriately, in its advanced stages is one of the major contributors to vision loss, irreversible blindness and visual impairment in older people worldwide (137–139). The main risk factors associated with AMD include age, gender, race, iris color, obesity, hypertension and genetics (140, 141). Presently, the AMD diagnosis includes dilated fundus examination along with optical coherence tomography and follow-up visits (142, 143). It has been shown that the activity of the complement system is strongly associated with the development of neovascular age-related macular degeneration, and that complement 2, complement 3 (C3) and complement 5 proteins play a major role in the classical complement pathway and pathogenesis of AMD (144). Therefore, some researchers have proposed a novel electrochemical C3 protein assay sensor for early detection of targeted drugs and monitoring of therapeutic efficacy in AMD patients (14). In exudative age-related macular degeneration, it has been suggested that the amount of vascular endothelial-derived growth factor (VEGF) in tears is related to vision loss, and for this reason, Kim et al. designed an electrochemical sensor for VEGF. Aids in the clinical diagnosis of AMD (145).

## 5 Future perspectives and conclusion

Since the introduction of the first glucose biosensor, biosensor technology in healthcare has developed by leaps and bounds. These biosensors are increasingly being used in a wide range of medical applications, providing physicians and patients with more convenient and accurate testing. With the global pandemic of COVID-19, the medical community’s need for non-invasive, fast and sensitive diagnostic methods has become even more urgent, and this has also led to a deep understanding that early screening and early diagnosis of diseases is an important means of maintaining one’s health. Compared with the invasive testing methods that require blood draws, the emergence of non-invasive electrochemical sensors has revolutionized medical testing by providing a safer, more convenient and affordable non-invasive early diagnostic method.

Tears are a fluid naturally secreted by the human body, and not only can they be accessed non-invasively, but they also contain biomarkers for a variety of diseases throughout the body, making them a highly promising diagnostic tool. Although tear sensors have been successfully used in the field of wearable biosensors, wearing contact lenses for long periods can cause adverse reactions such as inflammation and discomfort in the eye itself. In the future, while addressing the material and ocular adaptation of contact lenses, how to identify and validate these unique biomarkers to expand the scope of disease diagnosis, as well as to improve the accuracy and reproducibility of tear marker detection by electrochemical sensors are key issues that researchers need to address now, which include the analysis of biomarkers such as metabolites, proteins and nucleic acids. Addressing these issues will help tear sensors to be more widely used in diagnostic applications.

Overall, the research field of electrochemical biosensors for ocular applications has tremendous potential and opportunities. With the continuous progress of science and technology and the deepening of research, we have reason to believe that this field will revolutionize the diagnosis of eye diseases and the early screening and diagnosis of systemic diseases. This will not only greatly improve the accuracy and efficiency of medical diagnosis, but will also protect people’s health and bring a better future.

## Data Availability

Publicly available datasets were analyzed in this study. This data can be found at: https://webofscience.clarivate.cn/wos/alldb/basic-search.
